# Supplementary material for: Learning and diSentangling patient static information from time-series Electronic hEalth Records (STEER)
Source: PLOS Digit Health. 2024 Oct 21;3(10):e0000640. doi: 10.1371/journal.pdig.0000640 (PMC11493250; doi:10.1371/journal.pdig.0000640)
Supplement: S1 Text — (PDF) [file pdig.0000640.s001.pdf]

We applied either L1 or L2 regularization on FC models II, III, IV, V and VI (Figure 1a). We then analyzed the weights differences in these FC layers before and after regularization and compared them with the FC layers in the main branch (shown as the MLP block in Figure 1a).

When there is no regularization, the weights in block II to VI and block FC are similar (10^{-2} to 10^{-1}). After L1 regularization, weights in block II to block VI are all reduced to $\sim 10^{-4}$. After L2 regularization, block II to block V all have a significantly smaller weights (3 to 7 orders of magnitude) compared with no regularization. However, for FC layers weights in the main branch, changes are not noticeable after either regularization, as this is the step which is dealing with the time-series representations learned via the temporal blocks in the TCN model.

These results suggest a few possibilities: 1) The additional static information does not contain any useful information that is relevant to our target; 2) The FC layers may not be designed appropriately to integrate the information from the additional modality; and/or 3) The L1 and L2 regularization may be too strong, which can lead to overly small weights.

As for hypothesis 3, regularization is used to prevent overfitting. if it is too strong, it can cause the model to underfit and not make use of the additional modality. However, we examined the test set RMSE for L1 and L2 regularization, which are 1.70 and 1.69, respectively. This confirms that the regularization is not leading to underfitting.
