# Supplementary material for: Learning and diSentangling patient static information from time-series Electronic hEalth Records (STEER)
Source: PLOS Digit Health. 2024 Oct 21;3(10):e0000640. doi: 10.1371/journal.pdig.0000640 (PMC11493250; doi:10.1371/journal.pdig.0000640)
Supplement: S1 Fig — (PDF) [file pdig.0000640.s002.pdf]

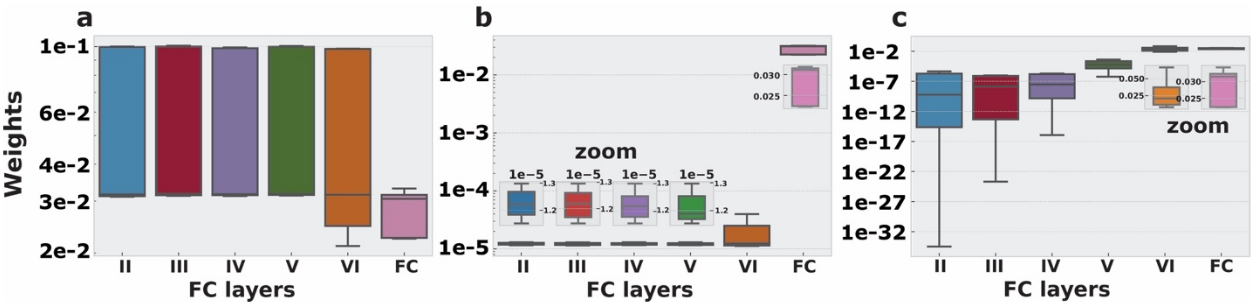


*Fig S1. Weights distribution box plot of fusion MLP layers from block II to VI and main model MLP layers. a) No regularization. b) L1 regularization. c) L2 regularization. Center line, median; box limits, upper and lower quartiles; whiskers, 1.5x interquartile range.*
