# Supplementary material for: Learning and diSentangling patient static information from time-series Electronic hEalth Records (STEER)
Source: PLOS Digit Health. 2024 Oct 21;3(10):e0000640. doi: 10.1371/journal.pdig.0000640 (PMC11493250; doi:10.1371/journal.pdig.0000640)
Supplement: S2 Fig — (PDF) [file pdig.0000640.s003.pdf]

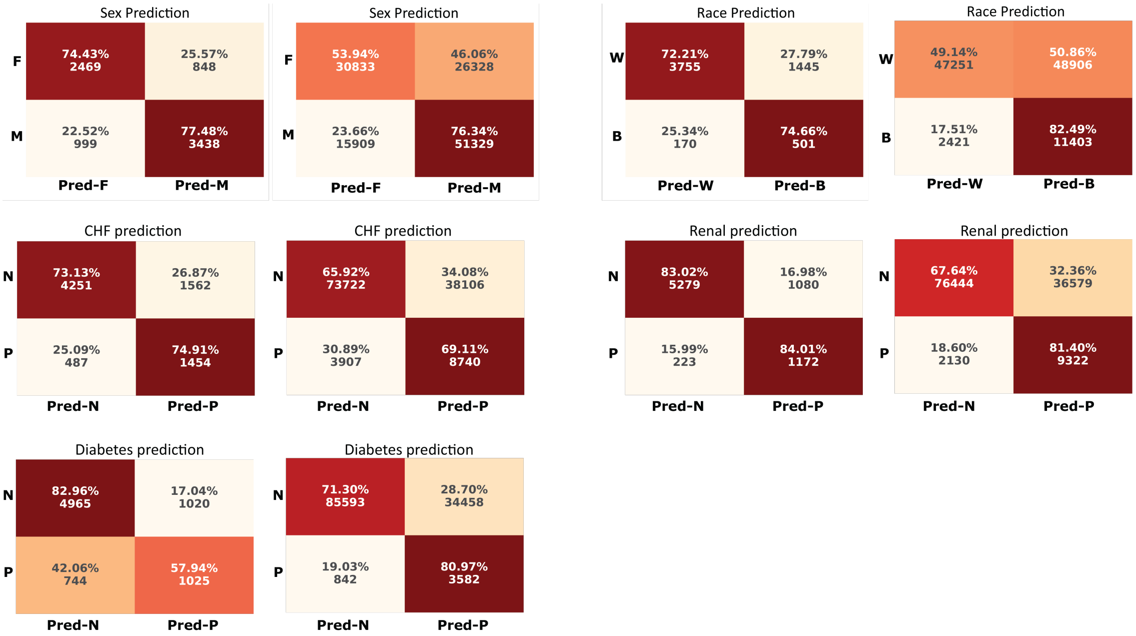

Fig S2. The confusion matrices on sex, race, CHF, renal disease, and diabetes. Left: prediction for MIMIC-IV test set. Right: the entire eICU records. F: Female. Pred-F: Predicted as Female. M: Male. Pred-M: Predicted as Male. W: White. Pred-W: Predicted as White. B: Black/African American. Pred-B: Predicted as Black/African American. N: Negative. Pred-N: Predicted as Negative. P: Positive. Pred-p: Predicted as Positive.
