# Supplementary material for: Learning and diSentangling patient static information from time-series Electronic hEalth Records (STEER)
Source: PLOS Digit Health. 2024 Oct 21;3(10):e0000640. doi: 10.1371/journal.pdig.0000640 (PMC11493250; doi:10.1371/journal.pdig.0000640)
Supplement: S1 Table — (PDF) [file pdig.0000640.s004.pdf]

Table S1. Predicting from original time-series variables, MIMIC-IV

	Sex	Age	Race	MI[1]	CHF[2]	PVD[3]	CBVD[4]	Dementia	CPD[5]
MIMIC-IV	0.857	0.876	0.833	0.781	0.833	0.704	0.813	0.876	0.695
eICU	0.740	0.789	0.770	0.677	0.754	0.590	0.794	0.766	0.728
	RD[6]	PUD[7]	MLD[8]	Diabetes	Paraplegia	Renal[9]	Cancer[10]	SLD[11]	MST[12]
MIMIC-IV	0.660	0.789	0.844	0.831	0.843	0.923	0.781	0.946	0.799
eICU	0.653	0.680	0.839	0.879	0.632	0.831	0.696	0.899	0.760

[1]MI: myocardial infarct. [2]CHF: congestive heart failure. [3]PVD: peripheral vascular disease. [4]CBVD: cerebrovascular disease. [5]CPD: chronic pulmonary disease. [6]RD: rheumatic disease.

[7]PUD: peptic ulcer disease. [8]MLD: mild liver disease. [9]Renal: renal disease.

[10]Cancer: malignant cancer. [11]SLD: sever liver disease. [12]MST: metastatic solid tumor.

The same acronyms are used for other SI tables.
