# Supplementary material for: Learning and diSentangling patient static information from time-series Electronic hEalth Records (STEER)
Source: PLOS Digit Health. 2024 Oct 21;3(10):e0000640. doi: 10.1371/journal.pdig.0000640 (PMC11493250; doi:10.1371/journal.pdig.0000640)
Supplement: S2 Table — (PDF) [file pdig.0000640.s005.pdf]

Table S1. Feature extraction model: LSTM, IHM

	Sex	Age	Race	MI	CHF	PVD	CBVD	Dementia	CPD
MIMIC-IV	0.830	0.860	0.799	0.746	0.786	0.670	0.788	0.847	0.677
eICU	0.667	0.767	0.762	0.672	0.680	0.569	0.801	0.741	0.720

	RD	PUD	MLD	Diabetes	Paraplegia	Renal	cancer	SLD	MST
MIMIC-IV	0.639	0.769	0.827	0.809	0.831	0.907	0.750	0.908	0.780
eICU	0.622	0.657	0.814	0.858	0.585	0.822	0.676	0.864	0.740
