# Supplementary material for: Learning and diSentangling patient static information from time-series Electronic hEalth Records (STEER)
Source: PLOS Digit Health. 2024 Oct 21;3(10):e0000640. doi: 10.1371/journal.pdig.0000640 (PMC11493250; doi:10.1371/journal.pdig.0000640)
Supplement: S3 Table — (PDF) [file pdig.0000640.s006.pdf]

Table S3. Feature extraction model: TCN, IHM

	Sex	Age	Race	MI	CHF	PVD	CBVD	Dementia	CPD
MIMIC-IV	0.827	0.855	0.798	0.748	0.816	0.681	0.786	0.842	0.688
eICU	0.701	0.762	0.745	0.676	0.750	0.575	0.799	0.726	0.722
	RD	PUD	MLD	Diabetes	Paraplegia	Renal	cancer	SLD	MST
MIMIC-IV	0.656	0.758	0.828	0.802	0.832	0.900	0.755	0.935	0.785
eICU	0.631	0.654	0.809	0.829	0.608	0.809	0.669	0.885	0.745
