# Supplementary material for: Learning and diSentangling patient static information from time-series Electronic hEalth Records (STEER)
Source: PLOS Digit Health. 2024 Oct 21;3(10):e0000640. doi: 10.1371/journal.pdig.0000640 (PMC11493250; doi:10.1371/journal.pdig.0000640)
Supplement: S4 Table — (PDF) [file pdig.0000640.s007.pdf]

*Table S4. Feature extraction model: Transformer, IHM*

| | Sex | Age | Race | MI | CHF | PVD | CBVD | Dementia | CPD |
|---|---|---|---|---|---|---|---|---|---|
| MIMIC-IV | 0.851 | 0.869 | 0.810 | 0.769 | 0.823 | 0.699 | 0.806 | 0.868 | 0.697 |
| eICU | 0.729 | 0.786 | 0.767 | 0.691 | 0.764 | 0.611 | 0.806 | 0.762 | 0.722 |
| | **RD** | **PUD** | **MLD** | **Diabetes** | **Paraplegia** | **Renal** | **cancer** | **SLD** | **MST** |
| MIMIC-IV | 0.653 | 0.777 | 0.836 | 0.814 | 0.848 | 0.908 | 0.772 | 0.941 | 0.798 |
| eICU | 0.634 | 0.661 | 0.826 | 0.870 | 0.619 | 0.830 | 0.675 | 0.883 | 0.766 |
