# Supplementary material for: Learning and diSentangling patient static information from time-series Electronic hEalth Records (STEER)
Source: PLOS Digit Health. 2024 Oct 21;3(10):e0000640. doi: 10.1371/journal.pdig.0000640 (PMC11493250; doi:10.1371/journal.pdig.0000640)
Supplement: S5 Table — (PDF) [file pdig.0000640.s008.pdf]

Table S5. Feature extraction model: LSTM, SOFA prediction, General cohort

	Sex	Age	Race	MI	CHF	PVD	CBVD	Dementia	CPD
MIMIC-IV	0.827	0.857	0.789	0.726	0.810	0.681	0.789	0.865	0.675
eICU	0.715	0.766	0.730	0.658	0.757	0.566	0.798	0.760	0.706
	**RD**	**PUD**	**MLD**	**Diabetes**	**Paraplegia**	**Renal**	**cancer**	**SLD**	**MST**
MIMIC-IV	0.644	0.780	0.813	0.792	0.809	0.914	0.744	0.925	0.764
eICU	0.623	0.666	0.820	0.836	0.588	0.825	0.652	0.888	0.732
