# Supplementary material for: Learning and diSentangling patient static information from time-series Electronic hEalth Records (STEER)
Source: PLOS Digit Health. 2024 Oct 21;3(10):e0000640. doi: 10.1371/journal.pdig.0000640 (PMC11493250; doi:10.1371/journal.pdig.0000640)
Supplement: S6 Table — (PDF) [file pdig.0000640.s009.pdf]

Table S6. Feature extraction model: TCN, SOFA prediction, General cohort

	Sex	Age	Race	MI	CHF	PVD	CBVD	Dementia	CPD
MIMIC-IV	0.840	0.862	0.807	0.750	0.817	0.692	0.785	0.878	0.683
eICU	0.721	0.772	0.743	0.675	0.744	0.571	0.793	0.754	0.707
	RD	PUD	MLD	Diabetes	Paraplegia	Renal	cancer	SLD	MST
MIMIC-IV	0.650	0.767	0.840	0.791	0.812	0.915	0.753	0.934	0.786
eICU	0.641	0.659	0.831	0.841	0.602	0.828	0.664	0.889	0.738
