# Supplementary material for: Learning and diSentangling patient static information from time-series Electronic hEalth Records (STEER)
Source: PLOS Digit Health. 2024 Oct 21;3(10):e0000640. doi: 10.1371/journal.pdig.0000640 (PMC11493250; doi:10.1371/journal.pdig.0000640)
Supplement: S7 Table — (PDF) [file pdig.0000640.s010.pdf]

Table S7. Feature extraction model: Transformer, SOFA prediction, General cohort

	Sex	Age	Race	MI	CHF	PVD	CBVD	Dementia	CPD
MIMIC-IV	0.845	0.860	0.807	0.744	0.819	0.688	0.794	0.867	0.683
eICU	0.725	0.760	0.767	0.658	0.766	0.565	0.812	0.772	0.714
	RD	PUD	MLD	Diabetes	Paraplegia	Renal	cancer	SLD	MST
MIMIC-IV	0.645	0.776	0.832	0.809	0.822	0.915	0.752	0.941	0.775
eICU	0.626	0.672	0.824	0.857	0.562	0.832	0.665	0.894	0.744
