# Supplementary material for: Learning and diSentangling patient static information from time-series Electronic hEalth Records (STEER)
Source: PLOS Digit Health. 2024 Oct 21;3(10):e0000640. doi: 10.1371/journal.pdig.0000640 (PMC11493250; doi:10.1371/journal.pdig.0000640)
Supplement: S8 Table — (PDF) [file pdig.0000640.s011.pdf]

Table S8. Feature extraction model: LSTM, SOFA prediction, Sepsis 3 cohort

	Sex	Age	Race	MI	CHF	PVD	CBVD	Dementia	CPD
MIMIC-IV	0.826	0.845	0.767	0.686	0.797	0.649	0.724	0.858	0.679
eICU	0.654	0.727	0.710	0.654	0.691	0.589	0.713	0.725	0.706
	**RD**	**PUD**	**MLD**	**Diabetes**	**Paraplegia**	**Renal**	**cancer**	**SLD**	**MST**
MIMIC-IV	0.562	0.700	0.840	0.765	0.752	0.889	0.718	0.923	0.782
eICU	0.630	0.580	0.778	0.789	0.605	0.790	0.697	0.849	0.748
