# Supplementary material for: Learning and diSentangling patient static information from time-series Electronic hEalth Records (STEER)
Source: PLOS Digit Health. 2024 Oct 21;3(10):e0000640. doi: 10.1371/journal.pdig.0000640 (PMC11493250; doi:10.1371/journal.pdig.0000640)
Supplement: S9 Table — (PDF) [file pdig.0000640.s012.pdf]

Table S9. Feature extraction model: TCN, SOFA prediction, Sepsis 3 cohort

	Sex	Age	Race	MI	CHF	PVD	CBVD	Dementia	CPD
MIMIC-IV	0.823	0.853	0.780	0.692	0.801	0.655	0.729	0.860	0.669
eICU	0.656	0.735	0.709	0.636	0.694	0.589	0.711	0.692	0.713
	**RD**	**PUD**	**MLD**	**Diabetes**	**Paraplegia**	**Renal**	**cancer**	**SLD**	**MST**
MIMIC-IV	0.619	0.739	0.844	0.770	0.666	0.896	0.724	0.919	0.769
eICU	0.652	0.582	0.771	0.766	0.639	0.795	0.715	0.837	0.762
