# Supplementary material for: Learning and diSentangling patient static information from time-series Electronic hEalth Records (STEER)
Source: PLOS Digit Health. 2024 Oct 21;3(10):e0000640. doi: 10.1371/journal.pdig.0000640 (PMC11493250; doi:10.1371/journal.pdig.0000640)
Supplement: S10 Table — (PDF) [file pdig.0000640.s013.pdf]

Table S10. Feature extraction model: Transformer, SOFA prediction, Sepsis 3 cohort

	Sex	Age	Race	MI	CHF	PVD	CBVD	Dementia	CPD
MIMIC-IV	0.831	0.852	0.778	0.709	0.802	0.675	0.736	0.850	0.673
eICU	0.668	0.722	0.741	0.656	0.686	0.581	0.730	0.700	0.734
	RD	PUD	MLD	Diabetes	Paraplegia	Renal	cancer	SLD	MST
MIMIC-IV	0.626	0.759	0.798	0.786	0.630	0.896	0.741	0.915	0.780
eICU	0.617	0.574	0.776	0.791	0.649	0.805	0.691	0.860	0.757
