# Supplementary material for: Learning and diSentangling patient static information from time-series Electronic hEalth Records (STEER)
Source: PLOS Digit Health. 2024 Oct 21;3(10):e0000640. doi: 10.1371/journal.pdig.0000640 (PMC11493250; doi:10.1371/journal.pdig.0000640)
Supplement: S11 Table — (PDF) [file pdig.0000640.s014.pdf]

*Table S11. eICU as the training database, test on MMIC-IV.*

| | Sex | Age | Race | CHF | Diabetes | Renal | SLD |
|---|---|---|---|---|---|---|---|
| eICU | 0.697 (0.693 -0.701) | 0.800 (0.796 - 0.804) | 0.764 (0.758 - 0.771) | 0.735 (0.729 - 0.741) | 0.804 (0.794 - 0.813) | 0.841 (0.836 - 0.846) | 0.878 (0.864 - 0.890) |
| MIMIC-IV | 0.736 (0.732 - 0.741) | 0.808 (0.805 - 0.812) | 0.721 (0.713 - 0.728) | 0.736 (0.732 - 0.741) | 0.692 (0.686 - 0.697) | 0.845 (0.841 - 0.849) | 0.884 (0.877 - 0.890) |
