# Supplementary material for: Learning and diSentangling patient static information from time-series Electronic hEalth Records (STEER)
Source: PLOS Digit Health. 2024 Oct 21;3(10):e0000640. doi: 10.1371/journal.pdig.0000640 (PMC11493250; doi:10.1371/journal.pdig.0000640)
Supplement: S12 Table — (PDF) [file pdig.0000640.s015.pdf]

Table S12. STEER results on eICU database

		STEER	Original
Sex	RMSE	1.86<br>(1.82 - 1.91)	1.84<br>(1.79 - 1.89)
	AUC	0.702<br>(0.671 - 0.734)	0.719<br>(0.702 - 0.738)
Age	RMSE	1.90<br>(1.86 - 1.95)	1.84<br>(1.79 - 1.89)
	AUC	0.787<br>(0.758 – 0.815)	0.815<br>(0.800 - 0.829)
Race	RMSE	1.95<br>(1.91 - 1.99)	1.84<br>(1.79 - 1.89)
	AUC	0.728<br>(0.681 - 0.776)	0.792<br>(0.767 - 0.815)
