# Supplementary material for: Learning and diSentangling patient static information from time-series Electronic hEalth Records (STEER)
Source: PLOS Digit Health. 2024 Oct 21;3(10):e0000640. doi: 10.1371/journal.pdig.0000640 (PMC11493250; doi:10.1371/journal.pdig.0000640)
Supplement: S13 Table — (PDF) [file pdig.0000640.s016.pdf]

*Table S13. STEER results on IHM task*

| | | STEER | Original |
|---|---|---|---|
| Sex | IHM AUC | 0.801 (0.761 - 0.839) | 0.879 (0.861 - 0.896) |
| | AUC | 0.806 (0.780 - 0.833) | 0.857 (0.854 - 0.860) |
| Age | IHM AUC | 0.821 (0.782 - 0.856) | 0.879 (0.861 - 0.896) |
| | AUC | 0.833 (0.808 - 0.859) | 0.876 (0.873 - 0.879) |
| Race | IHM AUC | 0.790 (0.748 - 0.832) | 0.879 (0.861 - 0.896) |
| | AUC | 0.734 (0.675 - 0.790) | 0.833 (0.828 - 0.838) |
