# Supplementary material for: Learning and diSentangling patient static information from time-series Electronic hEalth Records (STEER)
Source: PLOS Digit Health. 2024 Oct 21;3(10):e0000640. doi: 10.1371/journal.pdig.0000640 (PMC11493250; doi:10.1371/journal.pdig.0000640)
Supplement: S14 Table — (PDF) [file pdig.0000640.s017.pdf]

Table S14. Time-series variables

so2	po2	pco2	fio2
ph	base excess	bicarbonate	totalco2
hematocrit	hemoglobin	chloride	calcium
temperature	potassium	sodium	lactate
glucose	heart_rate	sbp	dbp
mbp	sbp_ni	dbp_ni	mbp_ni
resp_rate	wbc	basophils	eosinophils
lymphocytes	monocytes	neutrophils	atypical lymphocytes
bands	Immature granulocytes	metamyelocytes	nrbc
troponin_t	ck_mb	ntprobnp	albumin
total_protein	aniongap	bun	calcium
creatinine	fibrinogen	inr	pt
ptt	mch	mchc	mcv
platelet	rbc	rdw	screen
positive culture	has sensitivity	alt	alp
ast	amylase	bilirubin total	bilirubin direct
bilirubin indirect	ck cpk	ggt	ld_ldh
gcs	crp	weight	uo
central venous pressure	creatinine urine	magnesium	peak inspiratory pressure
phosphate	plateau pressure	positive end-expiratory pressure	positive end-expiratory pressure Set
red blood cell count urine	tidal volume observed	total protein urine	white blood cell count urine
pH urine	cul_site0	cul_site1	cul_site10
cul_site11	cul_site12	cul_site13	cul_site2
cul_site3	cul_site4	cul_site5	cul_site6
cul_site7	cul_site8	cul_site9	vent
antibiotic	dopamine	epinephrine	norepinephrine
phenylephrine	vasopressin	dobutamine	milrinone
heparin	crrt	rbc transfusion	platelets transfusion
ffp transfusion	colloid_bolus	crystalloid bolus	
