# Supplementary material for: Learning and diSentangling patient static information from time-series Electronic hEalth Records (STEER)
Source: PLOS Digit Health. 2024 Oct 21;3(10):e0000640. doi: 10.1371/journal.pdig.0000640 (PMC11493250; doi:10.1371/journal.pdig.0000640)
Supplement: Attachment — Submitted filename: Response_to_Reviewers.docx [file pdig.0000640.s018.docx]

Reviewer #1: This paper provides a comprehensive and thoroughly conducted study on the potential of time-series electronic health record data to forecast patient static information. The authors have made substantial contributions to the field of machine learning, and their findings are both original and influential. The methodology is strong and reliable, the analysis is thorough and extensive, and the findings are strongly supported by the data. Therefore, I recommend this paper for acceptance.

We thank Reviewer 1 for their endorsement of this work.

Reviewer #2: In the submitted manuscript titled "Learning and diSentangling patient static information from time-series Electronic hEalth Records (STEER)" the author suggest a method to protect patient-sensitive information using VAE model to diSentangle patient static information from Time-series Electronic hEalth Records (STEER) by composing a latent space of two subspaces, sensitive and non-sensitive.

Comments:

- How was the General and the Sepsis-3 cohorts defined?

Response 1: We agree with Reviewer 2 that this is important to specify. The definitions for both cohorts are in section 4.1 cohorts and features. To make this definition clearer to readers, we have added more details to it, as can be seen in the revised manuscript on page 16 line 310:

*“The General cohort is all the ICU stays in the MIMIC-IV and eICU databases whose age at ICU admission was 18 years or over and whose ICU stay was between 24h and 240h. As a result, we have 38,766 and 126,448 ICU records from these 2 databases, respectively. The second cohort, termed the Sepsis-3 cohort, is a subset of the General cohort which satisfies the Sepsis-3 criteria (32), in which organ dysfunction can be identified as an acute change in total SOFA score ≥2 points and the baseline SOFA score can be assumed to be zero in patients not known to have preexisting organ dysfunction. Therefore, out of the General cohort, we selected ICU stays that had SOFA score ≥2 anytime during the ICU stay and suspected infection to be the Sepsis-3 cohort.”*

- SOFA RMSE are 1.45-1.48 for the General cohort and 1.69-1.72 for the Sepsis-3 cohort, which are reasonable - A reasonable measure is not defined. Are there other publications with similar results? Or Clinical guidelines?

Response 2: We thank Reviewer 2 for bringing up this point. In terms of other related publications, there are many studies which are focused on building early warning systems for sepsis (Shashikumar et al. 2021), predicting sepsis onset (Moor et al. 2019), sepsis shock (Wardi et al. 2021) or sepsis-related mortality (Doorn et al. 2021) or treating the SOFA score prediction as a classification problem and only predicting its increase or decrease (Montomoli et al. 2021). The only one we found that is doing SOFA score regression prediction is from Aşuroğlu et al. 2021. Their SOFA score prediction MSE is 1.308. This is a bit smaller than our MSE, however, their prediction was only performed at 6 hours before sepsis onset, where the SOFA score itself is typically much lower since it is before the onset, and thus one would expect a smaller MSE. Our prediction is done for the entire ICU stay, and on average includes much higher SOFA scores, as can be observed in our added table below.

It is perhaps more informative to examine clinical guidelines. First, the SOFA score is an integer variable, with resolution of 1 (e.g. even if a patient's state is in between score 14 or 15, they won’t get 14.5). Additionally, clinical guidelines typically use a range of ~2 in SOFA score in their recommendations. For example, this foundational paper (Ferreira et al. 2001) in *JAMA* binned results in groups of 2, the Sepsis-3 guidelines (Singer et al. 2016) cite a change in SOFA of 2 in their sepsis diagnostic criteria. Many other examples exist. This forms the basis for our assessment that a SOFA score MSE between 1 and 2 is reasonable.

We have revised the text in the manuscript page 7 line 132 to:

*“Clinical guidelines typically use a range of ~2 in SOFA score in their recommendations, which forms the basis for our assessment that a SOFA score MSE between 1 and 2 is reasonable.”*

- Add to Table 1 summary statistics of SOFA.

Response 3: We have added the SOFA statistics for both cohorts in Table 1, reproduced below:

|  |  | General | Sepsis-3 |
| --- | --- | --- | --- |
| **SOFA** | 0 – 4:  4 – 8:  8 – 12:  12 – 16:  16 – 20:  20 – 24: | 56.40%  32.50%  8.64%  2.00%  0.42%  0.04% | 43.31%  39.96%  12.69%  3.28%  0.69%  0.06% |

From this table, we can also observe that the average SOFA score in General cohort is lower than the Sepsis-3 cohort, which is also consistent with lower RMSE we got for the General cohort.

- 48h mortality prediction - Do you predict anytime forward mortality, or was it limited to stays up to some certain length?

Response 4: We agree this is a very important point to consider. For 48h mortality prediction, we included all stays longer than 54h. This is because in our 48h in-hospital mortality prediction task, we incorporated a 6-hour gap between the end of the observation window (48h) and the mortality outcome to prevent the targeted task outcome leaking back into features. This is best practice as suggested in literature (Ghassemi et al. AMIA Summits Transl Sci Proc. 2020; Suresh H, Hunt N, Johnson A, Celi LA, Szolovits P, Ghassemi M. Clinical intervention prediction and understanding with deep neural networks. In: Machine Learning for Healthcare Conference. PMLR; 2017. p. 322–37). Thus, for example, if a stay is 51 hours long and the hospital_expire_flag is recorded true then we won’t consider that case.

We did actually perform in-hospital mortality prediction **without this 6 hour gap**, and not surprisingly the resulting AUC is higher: for TCN model, if we don’t use this gap, the AUC is 0.891 (0.859-0.922) and what we reported here in the manuscript is 0.879 (0.861 - 0.896). The results are expected since some “easy” cases are included if this gap is not considered, but that could result in a model that is not “actionable”. However, we include the 6h gap in our manuscript in order to make for a more realistic challenge.

We mentioned that we implemented the gap hour in our manuscript page 17 line 331, and we have now revised that text to make it clear that there is no upper limit in hospital stay:

*“Our first task is to predict IHM using 48h of ICU data. Besides, we also implemented a 6h gap between the end of the observation window (48h) and the positive outcome in order to prevent the targeted task outcome leaking back into features (45) and to support clinically actionable planning (46). Thus, we considered all stays of at least 54h in this analysis.”*

- SOFA prediction - is every hour of every patient considered as an observation in terms of training sample and test samples? Or was a random window selected to predict a single next SOFA of a patient? Were the patients/visits split to train and test without shared patients/visits?

Response 5: 1. Training sets and testing sets are definitely split **without shared visits** for both MIMIC-IV and eICU data to avoid any data leakage. 2. The time windows are not randomly selected, instead almost all time points are utilized. 3. Instead of predicting a single next SOFA, we are predicting a time-series SOFA.

Here is a schematic of our approach:

 

The model takes in **almost** an entire time series input and makes a time-series prediction. As can be seen from c). For example, if an ICU stay is 72 hours, data from 0 to 48h will be fed into the model, and the predictions are continuously for T=24h to 72h. What we need to ensure is that the prediction for T=24h is only dependent on T=0h (panel a above), prediction for T=25h if only dependent on T=0h and T=1h, …, and finally prediction for T=72h can depend on all the data from T=0h to 48h (panel c above). So we call our prediction 24h-later SOFA prediction due to the constant prediction horizon. In our main manuscript page 17 line 339, we have:

*“We used the multivariate time-series data to develop a model that iteratively gives the prediction for the patient’s future SOFA score. Let* $X=\left( X_{1}, X_{2}, X_{3}, {\ldots, X}_{T} \right)\in R^{m*T}$*, denote a multivariate time series, where m is the number of variables in the time series and* $X_{i}= \left( x_{i}^{1}, x_{i}^{2}, \ldots, x_{i}^{m} \right)$ *is the measurements of the input multivariate time series at time i. The prediction* $Y=\left( Y_{P+1}, Y_{P+2}, Y_{P+3}, {\ldots, Y}_{P+T} \right)$*, where* $Y_{P+1}$ *is the SOFA score at* $P+1$ *hour and is only dependent on* $X_{1}$*,* $Y_{P+2}$ *is dependent on both* $X_{1}, X_{2}$ *etc.. When making the final* $Y_{P+T}$*, the model is seeing the entire* $X=\left( X_{1}, X_{2}, X_{3}, {\ldots, X}_{T} \right)$*. We set the prediction horizon* $P$ *to be 24h in this study.”*

We agree that these points are critical, and we have thus added the following explanations connecting texts with the main figure in page 17 line 345:

*“The schematic of the SOFA score prediction strategy is shown in Figure 1a, where the input is the multivariate time series* $X$ *and the output is a predicted SOFA sequence. Each point* $Y_{P+T}$ *in the predicted sequence is only dependent on* $X=\left( X_{1}, X_{2}, X_{3}, {\ldots, X}_{T} \right).$*”*

- What were the features? This wasn't clear enough for both tasks.

Response 6: 1. Both tasks used the same set of time-series features to build the model and further to extract the learned representations. These time-series features include lab tests (platelets, creatinine etc.), vital signs (heart rate, respiratory rate etc.) as well as intervention features (ventilation, vasopressor etc.). In total there are 108 time-series features. Originally, we put the list of time-series features used in SI table S14. But we’ve made changes to make this clear in the main manuscript (described below).

2. Notably, despite using different model architectures and modeling cohorts to prove that our findings are generalizable, the same set of time-series features were used.

We’ve revised our manuscript based on this comment, which is on page 16 line 323.

*“The time-series features include lab tests (platelets, creatinine, lactate etc.), vital signs (heart rate, respiratory rate, blood pressure etc.) as well as intervention features. In total 108 time-series features were used. For details on the time-series features used, please refer to* …. *Despite using different model architectures and modeling cohorts to prove that our findings are generalizable, the same set of time-series features were used in all the studies conducted here.”*

- What are the 18 static features? There appears a list of only 7 in Table 3. I suggest you give the complete list in the Methods section, and don't rely on referring to the supplementary tables.

Response 7: Thanks for pointing this out! We only listed 7 of 18 in Table 3 for better readability. These 18 static features are: sex, age, race, myocardial infarct, congestive heart failure, peripheral vascular disease, cerebrovascular disease, dementia, chronic pulmonary disease, rheumatic disease, peptic ulcer disease, mild liver disease, diabetes, paraplegia, renal disease, malignant cancer, severe liver disease, metastatic solid tumor. We also added this list in the revised version, as can be seen on page 16 line 319:

*“The list of static features includes ….”*

We have the detailed results for the complete 18 static features in the supplementary tablet throughout Table S1 to S10, we have results for 1) predicting from original time-series variables 2-4) predicting from learned representations from 3 models for 48h in-hospital mortality prediction task, 5-7) predicting from learned representations from 3 models for SOFA prediction task, general cohort, 8-10) predicting from learned representations from 3 models for SOFA prediction task, sepsis-3 cohort.
